# Nutritional composition of two wild mushrooms consumed by the tribals of the Western Ghats of India

**DOI:** 10.1080/21501203.2014.917733

**Published:** 2014-05-19

**Authors:** Naga M. Sudheep, Kandikere R. Sridhar

**Affiliations:** a Department of Plant Science, School of Biological Sciences, Central University of Kerala, Riverside Campus, Padnekkad Nileswaram 671 328, Kerala, India; b Department of Biosciences, Mangalore University, Mangalagangotri, Mangalore 574 199, Karnataka, India

**Keywords:** edible mushrooms, *Agaricus abruptibulbus*, *Termitomyces globulus*, Western Ghat forests, nutritional composition, tribals

## Abstract

This study provides the nutritional qualities of two wild mushrooms (*Agaricus abruptibulbus* and *Termitomyces globulus*) commonly consumed by the tribals of Kaiga forests of the Western Ghats of India. Both mushrooms composed of high quantity of crude protein, crude fibre, calorific value and low quantity of crude lipid. Potassium and selenium contents were high, while sodium, calcium and phosphorus contents were low. Except for three essential amino acids (EAAs: leucine, tyrosine and lysine), the rest of the amino acids in both mushrooms were comparable to soybean and wheat. Based on the EAA standards of FAO-WHO, these mushrooms composed of high quantity of threonine, isoleucine and histidine. The EAA score of isoleucine in cooked *A. abruptibulbus* and threonine, isoleucine, phenylalanine, histidine and sulphur amino acids in cooked *T. globulus* were substantially high. Oleic acid constitutes a major unsaturated fatty acid in these mushrooms, which was significantly increased in cooked *A. abruptibulbus*. Cooking also increased the ratio of TUFA/TSFA in *A. abruptibulbus*, while it was opposite in *T. globulus*. Cooking significantly increased the linoleic acid in *A. abruptibulbus* and eicosadienoic acid in *T. globulus*.

## 1. Introduction

Macrofungi are the centre of attraction as food, medicine and cosmetics throughout the world (Halpern and Miller 2002; Boa 2004; Oboh and Shodehinde 2009; Hyde et al. 2010). Utilization of macrofungi as nutritional source (e.g. mushrooms, puffballs and morels) against plant and animal products is one of the viable avenues to fulfil the protein-energy demand (Boa 2004). According to a rough estimate, although over 2000 species of mushrooms occur worldwide, only 25 species have been widely accepted as food and a few species are successfully cultivated commercially (Lindequist et al. 2005). The proteins, essential amino acids (EAAs), fibre, minerals, fatty acids, vitamins and flavours of edible mushrooms are favourable for human nutrition and health (Sadler 2003; Oboh and Shodehinde 2009). Nutritional constituents of mushrooms are dependent on several factors like mushroom species, geographical region, substrate, stage of harvest and part of mushroom (Díez and Alvarez 2001; Sanmee et al. 2003; Barros et al. 2007b; Oboh and Shodehinde 2009). Although several surveys have been conducted on the diversity of mushrooms of the Western Ghats, very little is known on their nutritional status (e.g. Manoharachary et al. 2005; Swapna et al. 2008; Bhosle et al. 2010). The current study examines nutritional qualities of two wild mushrooms (*Agaricus abruptibulbus* and *Termitomyces globulus*) which are consumed by the tribals in Kaiga forests of the Western Ghats of India.

## 2. Materials and methods

### 2.1. Mushrooms and processing

*A. abruptibulbus* Peck and *T globulus* R. Heim & Gooss.-Font. were collected from the Kaiga forests adjacent to the Kali River of the Western Ghats of India (14°50′-14°51′ N, 74°24′–74°27′ E) during July-August 2010. *A. abruptibulbus* was gregarious on soil consisting of decomposing leaf litter underneath the bamboo culms, while *T. globulus* was common in and around the termite mounds. The mushrooms were identified based on their morphological features (Zoheri 1972; Purkayastha and Chandra 1985; Pegler and Vanhaecke 1994; Jordan 1995; Alexopolous et al. 1996; Phillips 2006; Mohanan 2011) and following herbarium specimens of the Department of Botany, Goa University, Goa, India.

Fresh weight of randomly sampled individual mushrooms (*n* = 25) was determined after blotting followed by oven drying (80°C) (Scientronic SBIM-25, New Delhi, India) to determine the moisture content gravimetrically. For nutritional analysis (proximal features, protein qualities and fatty acids), fresh blotted mushroom samples (*n* = 5) were grouped into two portions. The first portion of each replicate was oven dried (50–55°C) and the second portion was cooked in a household pressure cooker with distilled water (1:3 v/v) (6.5 L, Deluxe stainless steel, *TTK* Prestige™, Prestige Ltd., Hyderabad, India) followed by oven drying (50–55°C). The moisture content of uncooked and cooked flours was determined on oven drying (80°C, 24 hr). Dried uncooked and cooked mushrooms were milled (Wiley Mill, mesh #30), and the flours were refrigerated (4°C) in airtight containers for analysis.

### 2.2. Proximate and mineral analysis

The crude protein (N × 6.25) of the mushroom flours was estimated by micro-Kjeldahl method (Humphries 1956). The total lipid content was determined gravimetrically by extraction in petroleum ether using Soxhlet extractor (AOAC 1990). The crude fibre and ash contents were also gravimetrically determined (AOAC 1990). The carbohydrates were calculated according to Müller and Tobin (1980). The gross energy was determined based on Eknayake et al. (1999), while the total phenolics was determined using tannic acid standard (Rosset et al. 1982).

The mineral content (sodium, potassium, calcium, iron, copper, zinc, chromium, selenium, lead and nickel) was determined using atomic absorption spectrophotometry (GBC 932, Hampshire, IL, USA) (AOAC 1990). The vanadomolybdophosphoric acid method was employed to determine the total phosphorus using KH_2_PO_4_ as standard (AOAC 1990), and the ratios of Na/K and Ca/P were calculated.

### 2.3. Protein fractions, amino acids and protein digestibility

The mushroom flours were extracted twice with ice-cold trichloroacetic acid (TCA) to precipitate protein (Sadasivam and Manickam 1992), and the nitrogen in pooled supernatant was estimated by micro-Kjeldahl method (Humphries 1956) to determine the crude protein (N × 6.25). The total protein of mushroom flours was extracted according to Basha et al. (1976) and the precipitated protein in TCA was estimated based on Lowry et al. (1951). The ethanol treatment was omitted to save the prolamin fraction, albumin and globulin fractions were separated (Murray 1979), the supernatant containing glutelin was dissolved in alkali and protein fractions were precipitated in TCA and re-dissolved in NaOH to determine the protein content (Lowry et al. 1951).

The amino acids in mushroom flours were assessed by gas chromatography-combustion-isotope ratio mass spectrometry (Hewlett Packard 58,590 Series II gas chromatograph, Bremen, Germany) following the methods outlined by Hofmann et al. (1997, 2003) and Brand et al. (1994). The EAA/total amino acid (TAA) ratio was calculated.

The *in vitro* protein digestibility (IVPD) was determined according to Akeson and Stahmann (1964) using pepsin (Sigma, 3165 units mg^−1^ protein), trypsin (Sigma, 16,100 units mg^−1^ protein) and a-chymotrypsin (Sigma, 76 units mg^−1^ protein), and the EAA score was calculated.

### 2.4. Fatty acid analysis

The fatty acid methyl esters (FAMEs) of total lipids were prepared according to Pauda-Resurreccion and Banzon (1979). The gas chromatograph (GC-2010, Shimadzu, Japan) with auto injector (AOI), capillary column (BPX-70), analytical conditions of auto sampler, injection port settings, column oven settings and column information used for analysis of FAMEs were according to Nareshkumar (2007). The quantification of the FAMEs was performed using standard mixture (C4-C24) (Sigma-Aldrich, St. Louis, MO, USA) processed under similar conditions of samples. The concentration and area of each peak of FAME was computed using the GC post-run analysis software (Shimadzu, Japan).

### 2.5. Data analysis

The differences in proximate composition, minerals, protein fractions, amino acids, IVPD and FAMEs between the uncooked and cooked samples of mushrooms were assessed by *t*-test (Statistica version 8: StatSoft Inc. 2008).

## 3. Results and discussion

### 3.1. Proximate and mineral profiles

*A. abruptibulbus* and *T. globulus* possess desired traits like large size and biomass to consider as potential source of food. The moisture content of fresh mushrooms was higher in *A. abruptibulbus* (93.3 ± 0.24%, range 93.0–93.5%; *n* = 25) compared to *T. globulus* (91.8 ± 0.26%, range 91.5–91.9%; *n* = 25) and comparable to other species of agarics and termitomycetes (Kurtzman 1997; Gbolagade et al. 2006). As the high moisture content promotes microbial and enzyme activity, these mushrooms need precaution to prevent deterioration by freezing or drying immediately on sampling.

The moisture content of uncooked and cooked flours of mushrooms ranged between 4.2% and 7.6%. Although cooking significantly decreased the crude protein (20.3–23.8 vs. 18.3–19.5%) (Table 1), its content in cooked samples is comparable to edible legume seeds like green gram (*Phaseolus aureus*), pigeon pea (*Cajanus cajan*), chickpea (*Cicer arietinum*) and cowpea (*Vigna radiata* and *V. unguiculata*) (Khan et al. 1979; Jambunathan and Singh 1980; Nwokolo and Oji 1985; Nwokolo 1987). The protein content of *T. globulus* is lower (23.8 vs. 33.8%), while the crude fibre (9.7 vs. 3.7%), crude lipid (4.3 vs. 0.1%) and ash (17.7 vs. 13.9%) contents are higher than the *Termitomyces robustus* (Aletor and Aladetimi 1995). There was no significant increase in crude lipid between uncooked and cooked *A. abruptibulbus*, while it was significantly decreased in *T. globulus* (*p* < 0.05). The crude lipid content of the mushrooms is comparable with other edible mushrooms of India (1.9–4.3 vs. 0.6–4.7%) (Kavishree et al. 2008). The crude fibre (*p* < 0.01) was significantly increased in *A. abruptibulbus*, while its increase was not significant in *T. globulus*. Crude protein, crude lipid, crude fibre and ash contents of *A. abruptibulbus* are higher than other agarics (Kurtzman 1997). Based on the detailed analysis of edible mushrooms, Cheung (1997) opined that high quantity and unique composition of fibre have considerable importance in human nutrition and health. Even though low fibre diets are nutritionally appreciable in improving the digestibility by trapping less proteins and carbohydrates (Balogun and Fetuga 1986), the high fibre in diet has several health benefits as it lowers the blood cholesterol and reduces the risks associated with the large bowel cancer (Anderson et al. 1995; Slavin et al. 1997). Carbohydrates (*p* < 0.05) were significantly increased in *A. abruptibulbus*, while their increase was not significant in *T. globules*. The carbohydrates and calorific value of *A. abruptibulbus* and *T. globulus* were higher than many wild edible mushrooms (Colak et al. 2009). The high quantity of carbohydrates in the mushrooms studied is helpful to combat the intestinal cancer as well as inducing low glycaemic index to prevent the type II diabetes (Venn and Mann 2004). The total phenolics were significantly decreased on cooking in both mushrooms.

**Table 1.** Proximate composition of uncooked and cooked mushrooms on dry weight basis (*n* = 5; mean ± SD).

	*Agancus abruptibulbus*		*lermitomyces globulus*	
	Uncooked	Cooked	Uncooked	Cooked
Crude protein (g 100 g^−1^)	20.30 ± 0.94^a^	18.28 ± 0.71^b^*	23.83 ± 0.92^a^	19.54 ± 0.85^b^**
Crude lipid (g 100 g^−1^)	1.85 ± 0.80^a^	2.50 ± 0.10^a^	4.32 ± 0.46^a^	3.24 ± 0.43^b^*
Crude fibre (g 100 g^−1^)	8.85 ± 0.21^a^	9.52 ± 0.08^b^**	9.66 ± 0.30^a^	10.25 ± 0.28^a^
Ash (g 100 g^−1^)	19.70 ± 2.70^a^	10.17 ± 1.04^b^*	17.70 ± 1.40^a^	13.50 ± 2.02^a^
Carbohydrates (g 100 g^−1^)	49.31 ± 3.63^a^	59.53 ± 1.52^b^*	45.22 ± 2.85^a^	45.28 ± 3.13^a^
Calorific value (kJ 100 g”^1^)	1433 ± 50.15^a^	1593 ± 14.96^b^*	1559 ± 6.71^a^	1413 ± 36.34^b^*
Total phenolics (g 100 g^−1^)	2.29 ± 0.06^a^	1.68 ± 0.03^b^*	2.98 ± 0.11^a^	1.50±0.04^b^**

Note: Figures across the columns with different alphabets between uncooked and cooked mushrooms are significantly different (*t*-test: *p < 0.05, **p < 0.01).

Among the 10 minerals assessed in mushrooms, toxic metals like lead and nickel were below detectable level (Table 2). Unlike calcium, iron, copper and phosphorus contents, contents of sodium, potassium, chromium and selenium were significantly drained in cooked *A. abruptibulbus*, while the zinc content was not altered significantly. In *T. globules*, except for calcium, zinc, selenium and chromium contents, rest of the minerals showed similar trend like *A. abruptibulbus*. Among the minerals, mushrooms are known to possess high quantity of potassium (Dursun et al. 2006). In our study, both mushrooms possess high potassium and selenium contents, while sodium, calcium and phosphorus contents were low. Most of the minerals were considerably drained on cooking resulted in decreased ash content (Table 1). Sodium, potassium, calcium, iron and phosphorus contents of *A. abruptibulbus* were lower compared to other agarics (Kurtzman 1997), while sodium, potassium, calcium, iron, copper and zinc contents of *T. globulus* were higher than other termitomycetes (Gbolagade et al. 2006). Sodium, calcium and selenium contents of mushrooms were higher, while potassium (uncooked), iron, copper and zinc contents were lower than *Agaricus bisporus* and *Pleurotus sajor-caju* (Surinrut et al. 1987). Sodium and potassium contents were comparable, while calcium, iron, copper, zinc and phosphorus contents were below the dietary allowance for adults as recommended by NRC-NAS (1989). Increased selenium content in the mushrooms is in agreement with previous studies by Borovička and Řanda (2007). Increase in Na/K ratio was seen in cooked mushrooms, but the Ca/P ratio was increased in cooked *A. abruptibulbus* and decreased in *T. globulus*. The ratios of Na/K (0.03–0.07) and Ca/P (348–4026) of uncooked as well as cooked mushrooms were appreciable as foods with Na/K ratio <1 were known to control high blood pressure (Yusuf et al. 2007) and Ca/P ratio >1 prevents the loss of calcium in urine and restores calcium in bones (Shills and Young 1988). However, as caesium has been reported from the Kaiga environs (Karunakara et al. 2013), it is necessary to study the possibilities of accumulation of heavy metals as well as radioactive elements in the edible mushrooms and their potential risks on tribal health.

**Table 2.** Mineral composition of uncooked and cooked mushrooms (mg 100 g^−1^ dry mass) (*n* = 5; mean ± SD).

	*Agaricus abruptibulbus*		*Termitomyces globulus*				
	Uncooked	Cooked	Uncooked	Cooked	*Agaricus bisporus*^1^	*Pleurotus sajor-caju*^1^	Dietary allowance^2^
Sodium	171.34 ± 2.27^a^	74.27 ± 0.39^b^***	144.72 ± 3.32^a^	114.45 ± 4.63^b^**	26	87	120–200
Potassium	6059 ± 122^a^	2158 ± 201^b^***	3424 ± 13^a^	1675 ± 94^b^***	2640	3550	500–700
Calcium	152 ± 1.04^a^	765 ± 13.21^b^***	101 ± 1.04^a^	73 ± 0.81^b^***	19	45	600
Iron	0.04 ± 0.004^a^	0.10 ± 0.003^b^**	0.15 ± 0.002^a^	0.21 ± 0.02^b^*	8.84	23.90	10
Copper	0.12 ± 0.012^a^	0.19 ± 0.01^b^*	0.10 ± 0.003^a^	0.18 ± 0.02^b^*	0.62	4.20	0.6–0.7
Zinc	0.05 ± 0.004^a^	0.04 ± 0.004^a^	1.18 ± 0.03^a^	1.57 ± 0.10^b^*	6.81	13.10	5.0
Chromium	0.07 ± 0.08^a^	0.01 ± 0.003^b^**	0.01 ± 0.001^a^	0.01 ± 0.001^a^	—		—
Selenium	76.24 ± 0.38^a^	61.25 ± 0.51^b^***	70.54 ± 0.54^a^	33.89 ± 1.10^b^***	0.02	0.30	—
Lead	BDL	BDL	BDL	BDL	—	—	—
Nickel	BDL	BDL	BDL	BDL	—	—	—
Phosphorus	0.09 ± 0.01^a^	0.19 ± 0.004^b^***	0.13 ± 0.002^a^	0.21 ± 0.01^b^***	—	—	500
Na/K ratio	0.03	0.04	0.04	0.07	0.01	0.03	0.24–0.29
Ca/P ratio	1689	4026	777	348	—	—	1.2

Notes: Figures across the columns with different alphabets between uncooked and cooked mushrooms are significantly different (i-test: *p < 0.05, **p < 0.01, ***p < 0.001).

1 Surinrut et al. (1987).

2 NRC-NAS (1989) pattern for adults.

BDL, Below detectable level.

### 3.2. Protein qualities

The total protein was significantly decreased in cooked *A. abruptibulbus* (*p* < 0.05) as well as in *T. globulus* (*p* < 0.01) (Table 3). The albumin constitutes a major protein fraction followed by globulin in uncooked as well as in cooked mushrooms. These fractions were not significantly decreased in cooked mushrooms, so also the prolamin fraction as seen in leguminous seeds. As albumin consists of sulphur-amino acids including other EAAs (Baudoin and Maquet 1999), it has reflected in the presence of cystine, methionine and other EAA except for leucine and lysine in uncooked as well as in cooked mushrooms. There was no significant decrease of glutelin fraction in cooked *A. abruptibulbus* (*p* > 0.05), while cooking significantly decreased glutelin in *T. globulus* (*p* < 0.05). The non-protein nitrogen was also not significantly decreased in cooking mushrooms (*p* > 0.05).

**Table 3.** True protein, protein fractions and non-protein nitrogen of uncooked and cooked mushrooms (g 100 g^−1^ dry mass) (*n* = 5; mean ± SD).

		*Agaricus abruptibulbus*		*Termitomyces globulus*	
	Uncooked	Cooked	Uncooked	Cooked
True protein	19.18 ± 1.64^a^	16.19 ±2.66^b^*	21.75 ± 1.41^a^	17.17 ± 0.90^b^**
Albumin	7.76 ± 0.63^a^	6.82 ± 0.89^a^	9.83 ± 0.63^a^	8.17 ± 0.58^a^
Globulin	5.96 ± 0.86^a^	4.09 ± 1.17^a^	5.54 ± 1.34^a^	5.26 ± 1.55^a^
Prolamin	1.94 ± 0.24^a^	1.56 ± 0.34^a^	1.94 ± 0.24^a^	1.56 ± 0.34^a^
Glutelin	3.47 ± 0.67^a^	3.70 ± 1.35^a^	4.43 ± 0.46^a^	2.14 ± 0.89^b^*
Non-protein nitrogen	2.09 ± 0.83^a^	1.94 ± 0.34^a^	2.41 ± 0.23^a^	2.33 ± 0.59^a^

Note: Figures across the columns with different alphabets between uncooked and cooked mushrooms are significantly different (*t*-test: *p < 0.05, **p < 0.01).

Glutamic acid was the highest among the amino acids in uncooked as well as in cooked mushrooms (Table 4) and leucine, tyrosine and lysine were not detectable. Fourteen amino acids of uncooked *A. abruptibulbus* were significantly decreased on cooking, while only six amino acids (glutamic acid, serine, alanine, valine, isoleucine and tryptophan) were significantly decreased in *T. globulus*. No significant change was seen in aspartic acid, while six amino acids (threonine, proline, glycine, cystine, methionine, phenylalanine and histidine) were increased in cooked *T. globulus*. As seen in the present study, there are some instances of fluctuation of amino acids in *Agaricus bisporus* (Surinrut et al. 1987). The Alanine content in the fresh, dried and canned *A. bisporus* was 42.9, 54.4 and 49 g kg^−1^ protein, respectively. Similarly, in the fresh, dried and canned *A. bisporus*, phenylalanine content was 24.8, 24.9 and 37 g kg^−1^ protein and the methionine content was 15.6, 16.7 and 19.3 g kg ^1^ protein, respectively. Such increase in amino acids seems to be due to the impact of thermal treatment. Contents of glutamic acid, serine, threonine, proline, alanine, cystine and histidine were higher, while isoleucine, leucine, tyrosine, phenylalanine, tyrosine, lysine and arginine contents were lower and aspartic acid, glycine, valine and methionine contents were comparable to *Agaricus bisporus* and *Pleurotus sajor-caju* (Surinrut et al. 1987). With the exception of leucine, tyrosine, lysine and arginine, the quantity of rest of the amino acids is comparable to soybean (Bau et al. 1994) as well as to wheat (Pomeramz 1998).

**Table 4.** Amino acid composition of uncooked and cooked mushrooms in comparison with other food sources and FAO-WHO requirement pattern (g 100 g^−1^ protein; *n* = 5, mean ± SD).

	*Agaricus abruptibulbus*		*Termitomyces globulus*						
	Uncooked	Cooked	Uncooked	Cooked	*Agaricus bisporus*^1^	*Pleurotus sajor-caju*^1^	Soybean^2^	Wheat^3^	FAO-WHO^4^
EAA									
Threonine	5.91 ± 0.27^a^	4.32±0.17^b^**	6.25 ± 0.24^a^	10.47 ± 0.45^b^***	3.24	4.20	3.76	2.2–3	3.4
Valine	4.18 ± 0.19^a^	3.10±0.12^b^**	5.31 ± 0.21^a^	1.23 ± 0.05^b^***	2.86	4.39	4.59	3.7–1.5	3.5
Cystine	2.75 ± 0.13^a^	ND	0.845 ± 0.03^a^	4.37 ± 0.19^b^***	1.67	1.00	1.70	1.6–2.6	2.5^5^
Methionine	1.62 ± 0.08^a^	0.72 ± 0.03^b^***	0.68 ± 0.03^a^	5.45 ± 0.24^b^***	1.67	1.82	1.22	0.9–1.5	
Isoleucine	9.46 ± 0.44^a^	7.86±0.31^b^**	10.87 ± 0.42^a^	7.94 ± 0.34^b^***	2.29	7.59	4.62	3.4–1.1	2.8
Leucine	ND	ND	ND	ND	7.34	6.61	7.72	6.5–7.2	6.6
Tyrosine	ND	ND	ND	ND	2.29	3.01	1.24	1.8–3.2	6.3^6^
Phenylalanine	1.92 ± 0.09^a^	0.91 ± 0.04^b^***	2.73 ± 0.11^a^	4.83 ± 0.21^b^***	2.49	3.62	4.84	4.5–1.9	
Tryptophan	0.41 ± 0.02^a^	0.32±0.01^b^**	0.37 ± 0.01^a^	0.31 ±0.01^b^***	1.20	1.55	3.39	0.7–1	1.1
Lysine	ND	ND	ND	ND	4.51	3.96	6.08	1.8–2.4	5.8
Histidine	8.26 ± 0.38^a^	5.92 ± 0.23^b^**	5.21 ± 0.21^a^	11.59 ± 0.50 ^b^***	1.49	1.84	2.50	1.9–2.6	1.9
Non-EAA									
Glutamic acid	15.09 ± 0.71^a^	10.65 ± 0.42^b^**	21.11 ± 0.82^a^	14.71 ± 0.64^b^***	6.53	10.79	16.90	35.5–36.9	
Aspartic acid	10.98 ± 0.51^a^	9.06 ± 0.35^b^**	8.69 ± 1.07^a^	7.37 ± 0.32^a^	7.03	7.45	11.30	3.7–4.2	
Serine	5.62 ± 0.26^a^	4.45±0.17^b^**	5.18 ± 0.20^a^	4.79 ± 0.21^b^*	3.32	7.02	5.67	3.7–4.8	
Proline	6.21 ± 0.29^a^	4.21 ±0.16^b^**	5.66 ± 0.22^a^	9.1 ± 0.39^b^***	ND	ND	4.86	11.4–11.7	
Alanine	7.15 ± 0.33^a^	4.93 ± 0.19^b^**	10.12 ± 0.39^a^	6.69 ± 0.29^b^***	5.44	6.40	4.23	2.8–3	
Glycine	3.21 ± 0.15^a^	2.30 ± 0.09^b^**	2.85 ± 0.11^a^	8.32 ± 0.36^b^***	2.95	4.50	4.01	3.2–3.5	
Arginine	ND	ND	ND	ND	3.76	3.94	7.13	3.1–3.8	
EAA/TAA ratio	0.417	0.394	0.376	0.475	0.517	0.497	0.435	0.353–0.628	

Notes: Figures across the columns with different alphabets between uncooked and cooked mushrooms are significantly different (*t*-test: *p < 0.05, **p < 0.01, ***p < 0.001).

1 Surinrut etal. (1987).

2 Bau et al. (1994).

3 Pomeramz (1998).

4 FAO-WHO (1991).

Cystine + methionine.

Tyrosine + phenylalanine.

EAA, Essential amino acid; TAA, Total amino acid; ND, Not detectable.

On comparison with FAO-WHO (1991) EAA reference pattern for adults, both mushrooms possess higher quantity of threonine, isoleucine and histidine, but the rest of EAAs were lower or devoid of them in mushrooms, with a few exceptions. For instance, some EAAs were high in uncooked *A. abruptibulbus* (valine and cysteine + methionine) and uncooked (valine) or cooked (cysteine + methionine and phenylalanine) *T. globulus*. Except for leucine and lysine, the EAA score of threonine, isoleucine and histidine was high in uncooked and cooked mushrooms. However, the EAA score of isoleucine in cooked *A. abruptibulbus* and threonine, cysteine + methionine, isoleucine, phenylalanine and histidine in cooked *T. globulus* was considerably high. The EAA/TAA ratio was higher in uncooked than in cooked *A. abruptibulbus*, while it was reverse in *T. globulus*. The ratio of EAA/TAA of both mushrooms was higher than that of *A. bisporus* and *P. sajor-caju*, while comparable to soybean (Bau et al. 1994) and wheat (Pomeramz 1998).

In addition to amino acids, *in vivo* protein digestibility or IVPD is a valuable index to ascertain the quality of proteins. Cooking significantly elevated the IVPD in mushrooms (Table 5), revealing elimination or inactivation of antinutritional factors and improvement of nutritional qualities. The total phenolics of uncooked mushrooms was also decreased significantly on cooking. However, phenolics and tannins in small quantities in food stuffs will be beneficial due to their antioxidant properties (Hertog et al. 1997; Hagerman et al. 1998; Oboh and Shodehinde 2009). According to Barros et al. (2007b), phenolics and flavonoids of three wild edible mushrooms serve as antimicrobial agents against pathogenic bacteria (e.g. *Bacillus cereus*) and fungi *{Candida albicans* and *Cryptococcus neoformans)*. It is interesting to note that the quantities of polyphenols varied depending on the part of mushroom, which was higher in pileus than in stipe (of *Termitomyces robustus, Coprinus* sp. and *Volvariella escu-lenta*), but the free radical scavenging activity was higher in stipe compared to pileus (Oboh and Shodehinde 2009). Similar observations were made by Barros et al. (2007b) on the polyphenols, flavonoids, ascorbic acid, β-carotene and lycopene.

**Table 5.** *In vitro* protein digestibility (TVPD) (*n* = 5; mean ± SD) and essential amino acid (EAA) score in uncooked and cookec mushrooms.

	*Agaricus abruptibulbus*		*Termitomyces globulus*						
	Uncooked	Cooked	Uncooked	Cooked
IVPD (%)	62.81 ± 8.62^a^	81.46 ±2.29^b^*	47.91 ± 8.15^a^	75.41 ± 7.63^b^**
EAA score (%)				
Threonine	173.8	127.3	184.0	307.1
Valine	119.4	88.6	151.7	35.3
Cystine + methionine	181.6	28.7	61.1	392.8
Isoleucine	337.9	280.7	388.2	283.6
Leucine	ND	ND	ND	ND
Tyrosine + phenylalanine	30.4	14.1	45.0	76.6
Tryptophan	35.9	29.4	33.8	28.5
Lysine	ND	ND	ND	ND
Histidine	434.7	311.6	182.1	610.2

Notes: Figures across the columns with different alphabets between uncooked and cooked mushrooms are significantly different (*t*-test: *p < 0.05, **p < 0.01).

ND, Not detectable.

The EAA score of threonine, isoleucine and histidine was high in uncooked as well as in cooked mushrooms. The EAA score of cysteine + methionine and tyrosine + phenylalanine of *T. globulus* showed increase in cooked samples, but the score was decreased in rest of the EAAs.

### 3.3. Fatty acid profile

Among the saturated fatty acids, palmitic acid was the major fatty acid in uncooked mushrooms (Table 6). Unlike *T. globulus* (*p* < 0.05), the palmitic acid decreased drastically in cooked *A. abruptibulbus* (*p* < 0.01). Oleic acid was the major fatty acid among the unsaturated fatty acids in uncooked mushrooms. Oleic acid increased significantly in cooked *A. abruptibulbus* (*p* < 0.01), while it was significantly decreased in cooked *T. globulus* (*p* < 0.01). Predominance of oleic and palmitic acids in mushrooms studied is in agreement with the reports on other mushrooms in India (Longvah and Deosthale 1998; Kavishree et al. 2008) as well as in Northeast Portugal (Barros et al. 2007a). Interestingly, two essential fatty acids like linoleic acid (*A. abruptibulbus*) (*p* < 0.05) and eicosadienoic acid (*T. globulus*) (*p* < 0.01) of mushrooms showed significant increase on cooking. The total saturated fatty acids (TSFAs) decreased (*p* < 0.01) in cooked *A. abruptibulbus*, while it was reverse in *T. globulus* (*p* < 0.01), and the content of total unsaturated fatty acids (TUFAs) was opposite to TSFA.

**Table 6.** Fatty acid methyl esters (g kg^−1^ dry mass) of uncooked and cooked mushrooms (*n* = 5, mean ± SD).

	*Agaricus abruptibulbus*		*Termitomyces globulus*		
	Uncooked	Cooked	Uncooked	Cooked
Saturated fatty acids				
Lauric acid (C12:0)	1.50 ± 0.46	—	0.06 ± 0.01	—
Myristic acid (C14:0)	0.73 ± 0.22	—	0.06 ± 0.01^a^	0.13 ± 0.02^b^**
Palmitic acid (C16:0)	7.02 ± 2.14^a^	1.98 ± 0.07^b^**	10.58 ± 1.13^a^	8.88 ± 1.21^b^*
Heptadecanoic acid (C17:0)	—	—	0.08 ± 0.02	—
Stearic acid (C18:0)	2.20 ± 0.67	—	—	12.28 ± 1.66
Arachidic acid (C20:0)	—	0.15 ±0.01	—	0.61 ± 0.08
Behenic acid (C22:0)	0.92 ± 0.28^a^	0.05 ± 0.002^b^*	0.09 ± 0.01^a^	0.21 ± 0.03^b^**
Tricosanoic acid (C23:0)	—	—	0.04 ± 0.004	—
Unsaturated fatty acids				
Palmitoleic acid (C16:1)	1.35 ± 0.41^a^	0.05 ± 0.002^b^*	0.11 ± 0.01^a^	0.32 ± 0.04^b^**
Oleic acid (C18:l)	1.40 ± 0.42^a^	3.06 ± 0.94^b^**	16.95 ± 1.82^a^	7.50 ± 1.01^b^**
Linoleic acid (C18:2)	0.71 ± 0.22^a^	0.87 ± 0.03^b^*	—	—
Elaidic acid (C18:1)	—	—	11.42 ± 1.22	—
Eicosenoic acid (C20:l)	—	—	0.48 ± 0.03	—
Eicosadienoic acid (C20:2)	—	—	0.16 ± 0.02^a^	0.31 ± 0.04^b^**
Nervonic acid (C24:l)	—	0.06 ± 0.002	0.06 ± 0.01^a^	0.12 ± 0.02^b^*
Total saturated fatty acids (TSFAs)	12.38 ± 3.77^a^	2.18 ± 0.08^b^**	10.91 ± 1.17^a^	21.06 ±3.01^b^**
Total unsaturated fatty acids (TUFAs)	3.46 ± 1.04^a^	4.04 ± 0.03^b^*	29.17 ± 3.19^a^	8.25 ± 1.10^b^**
TUFA/TSFA ratio	0.28	1.85	2.67	0.39

Note: Figures across the columns with different alphabets between uncooked and cooked mushrooms are significantly different (*t*-test: *p < 0.05, **p < 0.01).

## 4. Conclusions

This study demonstrated that cooked *A. abruptibulbus* and uncooked *T. globulus* were endowed with potential nutritional principles. Evaluation of nutritional component (proteins, fibre, carbohydrates, minerals, amino acids, unsaturated fatty acids and IVPD) and calorific value of mushrooms clearly demonstrated low fat and low calorie diets, which fall between legumes and meat (FAO-WHO 1989). As IVPD was higher in cooked mushrooms, uncooked/partially cooked/cooked mushrooms could be utilized depending on the nutritional requirement of a specific product. Wide distribution of *A. abruptibulbus* especially in the soils underneath bamboo culms and gregarious occurrence of *T. globulus* in and around the termite mounds of Kaiga forests show prevalence of suitable ecological and edaphic factors (e.g. soil qualities, soil moisture, leaf litter qualities and organic matter) for their perpetuation. As a variety of medicinally valued compounds have been isolated from the wild and cultivated mushrooms of the Asian origin, *A. abruptibulbus* and *T. globulus* of the Western Ghats may serve as potential sources of nutraceuticals. Further attempts are necessary for commercial cultivation and evaluation of bioactive potential of different parts of these mushrooms.
